# High density lipoproteins mediate in vivo protection against staphylococcal phenol-soluble modulins

**DOI:** 10.1038/s41598-021-94651-1

**Published:** 2021-07-28

**Authors:** Josefien W. Hommes, Rachel M. Kratofil, Sigrid Wahlen, Carla J. C. de Haas, Reeni B. Hildebrand, G. Kees Hovingh, Micheal Otto, Miranda van Eck, Menno Hoekstra, Suzanne J. A. Korporaal, Bas G. J. Surewaard

**Affiliations:** 1 Department of Microbiology, Immunology, and Infectious Disease. Snyder Institute for Chronic Diseases, University of Calgary, Calgary, AB Canada; 2 Medical Microbiology, University Medical Center Utrecht, Utrecht, The Netherlands; 3 Department of Diagnostic Sciences, Laboratory of Experimental Immunology, Ghent University, Ghent, Belgium; 4 Division of BioTherapeutics, Leiden Academic Centre for Drug Research, Gorlaeus Laboratories, Leiden, The Netherlands; 5 Department of Vascular Medicine, Academic Medical Center, Amsterdam, The Netherlands; 6 Pathogen Molecular Genetics Section, Laboratory of Bacteriology, National Institute of Allergy and Infectious Diseases, National Institutes of Health, Bethesda, MD USA; 7 Department of Clinical Chemistry and Haematology, University Medical Center Utrecht, Utrecht, The Netherlands

**Keywords:** Infection, Inflammation, Infectious diseases, Bacterial infection, Infectious diseases, Innate immunity, Immunology, Microbiology

## Abstract

*Staphylococcus aureus* virulence has been associated with the production of phenol-soluble modulins (PSMs). These PSMs have distinct virulence functions and are known to activate, attract and lyse neutrophils. These PSM-associated biological functions are inhibited by lipoproteins in vitro. We set out to address whether lipoproteins neutralize staphylococcal PSM-associated virulence in experimental animal models. Serum from both LCAT an ABCA1 knockout mice strains which are characterised by near absence of high-density lipoprotein (HDL) levels, was shown to fail to protect against PSM-induced neutrophil activation and lysis in vitro. Importantly, PSM-induced peritonitis in LCAT^−/−^ mice resulted in increased lysis of resident peritoneal macrophages and enhanced neutrophil recruitment into the peritoneal cavity. Notably, LCAT^−/−^ mice were more likely to succumb to staphylococcal bloodstream infections in a PSM-dependent manner. Plasma from homozygous carriers of ABCA1 variants characterized by very low HDL-cholesterol levels, was found to be less protective against PSM-mediated biological functions compared to healthy humans. Therefore, we conclude that lipoproteins present in blood can protect against staphylococcal PSMs, the key virulence factor of community-associated methicillin resistant *S. aureus*.

## Introduction

*Staphylococcus aureus (S. aureus)* is a commensal organism in humans and about 30% of healthy individuals are colonized asymptomatically with *S. aureus* on the skin and in the nasopharyngeal cavity^1^. Occasionally, *S. aureus* can spread from the nostrils and cause infections ranging from superficial skin infections to potential fatal endocarditis, sepsis or necrotizing fasciitis^2,3^. *S. aureus* is notorious for its resistance to antibiotics and especially methicillin-resistant *S. aureus* (MRSA) is of major clinical concern, as it is among the leading causes of death by bacterial infections in the western world^4^. Community-associated (CA-)MRSA strains are generally regarded as more virulent than hospital-associated (HA-)MRSA, because they can cause infections in otherwise healthy individuals^5,6^.

The success of MRSA in the human host can be attributed to its wealth of virulence factors, as these factors control many aspects of its commensal and pathogenic lifestyle. Among the many virulence factors and immune evasion molecules described for MRSA^7^, phenol-soluble modulins (PSMs) are one of the few virulence factors contributing to the success of CA-MRSA strains^8,9^. PSMs are small cytolytic core genome-encoded peptide toxins, of which the expression is strictly controlled by the accessory gene regulator (*agr*) system^10^. These cytolytic peptides are among the highest produced peptides in an overnight culture and can account for 60% of the total protein production. Although PSMs share a common amphipathic α-helical region, which is thought to enable their cell lytic ability by disrupting the cell membrane, PSMs are categorized in two groups, based on their size^9^. The smaller α-type PSM group, consisting of four PSMα peptides and δ-toxin with a length of 20–30 amino acids, are more cytolytic and proinflammatory^9^ compared to the larger 44 amino acids β-type PSMs. In addition, within the *SSCmec* locus of MRSA strain belonging to the SCC*mec* types II, IIA, IIB, IID, III, and VIII, an additional α-type PSM was found named PSMmec^11^. Isogenic PSMα mutants in different genetic backgrounds show reduced virulence in bacteremia and skin infection models compared to their wildtype CA-MRSA strains in both mice and rabbits^9,12^.

Next to their cytolytic potential towards host cells including neutrophils, PSMs can stimulate neutrophils directly via formylated peptide receptor 2 (FPR2)^13^. Activation of this G protein-coupled receptor expressed on neutrophils, monocytes, macrophages, immature dendritic cells, and microglial cells induces chemotaxis, degranulation and superoxide generation^14^. While micromolar concentrations of PSMs are needed for neutrophil lysis, nanomolar concentrations are enough for FPR2-mediated neutrophil stimulation^13^.

Most studies that investigated PSM function in vitro have used culture media without the addition of serum^9,11,13,15,16^. However, our group discovered that PSMs are functionally inhibited by lipoproteins that are abundantly present in serum and other body fluids^17^. Lipoproteins perform a key role in physiology by transporting lipids to and from the liver. Lipoproteins are complex particles with a neutral core containing triglycerides and cholesterol-esters, covered by an amphipathic monolayer of phospholipids and unesterified cholesterol and an apolipoprotein that binds and stabilizes the particles^18^. Reverse cholesterol transport is a pathway that describes the high density lipoprotein (HDL)-mediated removal of excess cholesterol from peripheral cells to the liver for excretion by the bile^19–21^. Both lecithin cholesterol acyltransferase (LCAT)^22,23^ and ATP-binding cassette transporter A1 (ABCA1)^20,24^ play a central role in this process. ABCA1 is involved in the formation of lipid-poor apoA-I into pre-β-HDL, a discoidal particle that consists of phospholipids and apoA-I and stimulates the release of cholesterol. LCAT converts the cholesterol accepted by lipid-poor apoA-I into cholesteryl esters and transforms pre-β HDL into a small spherical HDL particle. ABCA1^−/−^ mice are characterized by plasma HDL deficiency^25^, whereas LCAT^−/−^ mice only have non-mature, cholesterol poor, pre-β-HDL particles in their plasma^26^. Current evidence for the functional inhibition of PSMs by serum lipoproteins was obtained mainly by in vitro studies*,* whether PSM neutralization occurs in vivo remains to be investigated^17^. Here, we extended this line of investigation by mice deficient for LCAT and ABCA to evaluate the role of HDL lipoproteins in various animal models of sterile inflammation and staphylococcal infection. This study shows that HDL particles can efficiently scavenge PSM peptides produced by *S. aureus* during infection and prevent host cellular damage.

## Results

### Human and mouse plasma neutralize PSMs

**Figure 1:**
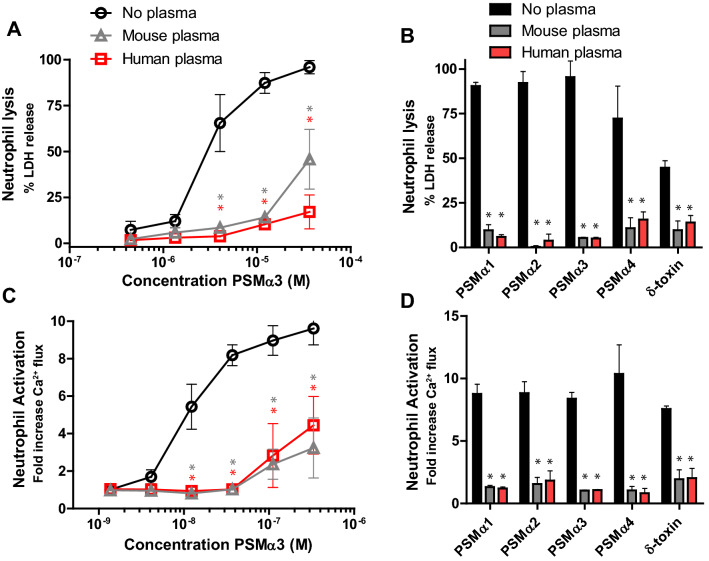
Human and murine control plasma inhibit PSM-mediated neutrophil lysis and activation. (**A**) Dose-dependent neutrophil lysis by synthetic PSMα3 (400 nM to 30 µM), preincubated with or without 1% human or murine plasma. (**B**) Neutrophil lysis by synthetic PSMα1-3 (10 µM), PSMα4 and δ-toxin (50 µM) pretreated with or without 1% human or mouse plasma. Neutrophil lysis was measured through LDH release. (**C**) Dose–response curves for calcium mobilization in HL-60/FPR2 cells induced by PSMα3 with 0.1% human or mouse plasma-treated PSMα3. (**D**) Calcium mobilization of HL-60/FPR2 stimulated with 50 nM PSMα1, 500 nM PSMα2, 50 nM PSMα3, 5 µM PSMα4, and 200 nM δ-toxin, all pre-incubated with or without 0.1% human or mice plasma, before calcium mobilization was measured by flow cytometry. Red and gray * indicates statistical significance of p < 0.01 to no plasma conditions. Data represent means ± SEM of 3–5 independent experiments. One-way ANOVA.

First, we evaluated whether murine plasma inhibit the biological functions of PSMs to an equal extent as previously shown in experiments where human plasma was used^17^. Indeed, murine plasma inhibited the PSMα3-induced lysis of neutrophils as potently as human plasma (Fig. 1A). Besides PSMα3, the most active α-type PSM^9^, *S. aureus* PSMα1, PSMα2, PSMα4 and δ-toxin were tested at lytic concentrations. Again, murine plasma was equally effective as human plasma and completely abrogated the lysis of human neutrophils induced by the synthetic PSM peptides (Fig. 1B). Many staphylococcal toxins, such as leukocidins display strict host and cell-type specificity. Typically, these leukocidins bind to cellular receptors before oligomerization into pores, which has helped to explain the cellular tropism and species selectivity of these toxins^27^. The G-protein coupled receptor FPR2, that is abundantly expressed on phagocytes, can detect nanomolar levels of PSM peptides, however FPR2 is not required for PSM-induced host cell lysis. Subsequently, PSMs have not been found to display species selectivity for their lytic capacity, as we observed no difference in susceptibility to PSM intoxication between human and mouse neutrophils (Supplementary Fig. 1). Next, we analyzed Ca^2+^ flux as a general measure to test for neutrophil activation, as used in previous studies^13,17^. HL60 cells, a neutrophil-like cell line, stably transfected with the FPR2 gene was used to determine the effect of murine plasma on PSM-induced neutrophil activation via FPR2. Both human and murine plasma comparably reduced the neutrophil-activating capacity of PSMα3 throughout a range of PSMα3 concentrations (Fig. 1C). Moreover, *S. aureus* PSMα1, PSMα2, PSMα4 and δ-toxin were significantly inhibited by murine and human plasma in their potency to activate neutrophils via Ca^2+^ flux (Fig. 1D). These data show that similar to human plasma, murine plasma can functionally inhibit the important virulence factor PSMs produced by *S. aureus*.

### Mice with lower plasma HDL levels fail to neutralize PSMs

**Figure 2:**
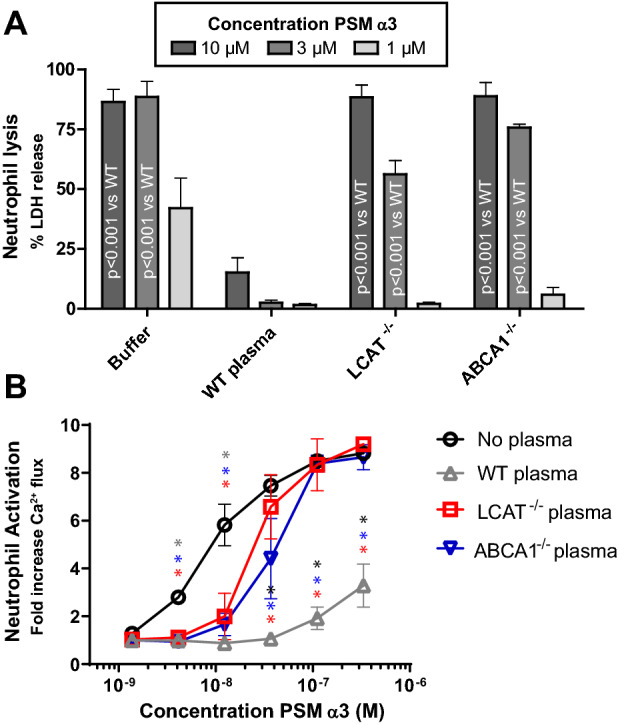
Reduced protection against PSM-mediated neutrophil activation and lysis by plasma from ABCA1^−/−^ or LCAT^−/−^ mice. (**A**) Dose-dependent human neutrophil lysis by synthetic PSMα3 (10, 3 and 1 µM), preincubated with 0.5% plasma derived from WT(C57BL/6), ABCA1^−/−^ or LCAT^−/−^ mice. Neutrophil lysis was measured through LDH release. Data represent means ± SEM of 4 independent experiments. (**B**) Dose–response curves for calcium mobilization in HL-60/FPR2 cells induced by PSMα3 preincubated with 0.1% plasma from WT, ABCA1^−/−^ or LCAT^−/−^ mice. * indicates statistical significance of p < 0.01. At concentrations below 10 nM statistical significance was found between no plasma conditions and pretreatment with WT, ABCA1^−/−^ or LCAT^−/−^. Above 30 nM PSMα3 WT plasma was significantly different from ABCA1^−/−^, LCAT^−/−^ or no plasma. Data represent means ± SEM of 4–5 independent experiments.

HDL particles have been shown to functionally inhibit PSM-mediated functions in vitro^17^. To evaluate whether experimental mouse models would be feasible, we performed in vitro experiments with plasma from genetically modified mice models with reduced HDL cholesterol levels ABCA1^−/−^^24^ and LCAT^−/−^ mice^28^. As expected, plasma from ABCA1^−/−^ and LCAT^−/−^ mice was unable to inhibit a challenge with lytic concentrations of PSMα3 compared to wildtype (WT) plasma (Fig. 2A). At 0.5% v/v, WT plasma showed a near-complete inhibition of PSMα3-induced lysis, whereas plasma from ABCA1^−/−^ and LCAT^−/−^ mice failed to protect neutrophils from lysis at 10 µM and 3 µM PSMα3 concentration (Fig. 2A). Notably, at 1 µM PSMα3, there was still protection from lysis by ABCA1^−/−^ and LCAT^−/−^ plasma compared to buffer conditions without plasma, presumably mediated through other lipoprotein fractions in the plasma, which are not affected by the genetic deletion of ABCA1 and LCAT^24,28^.

Furthermore, by testing the plasma derived from ABCA1^−/−^ and LCAT^−/−^ mice, we evaluated the role of HDL in protection against PSMα3-induced neutrophil activation. At concentrations higher than 30 nM PSMα3, ABCA1^−/−^ and LCAT^−/−^ plasma was unable to inhibit neutrophil Ca^2+^ flux, while WT plasma showed potent inhibition (Fig. 2B). Similarly, as observed for PSM-induced lysis, ABCA1^−/−^ and LCAT^−/−^ plasma displayed inhibitory capacity at low PSM concentrations. In our hands, ABCA^−/−^ mice were difficult to breed, so for any subsequent experiments we only used LCAT^−/−^ mice. Altogether, this data shows that plasma HDL can potently inhibit PSM-induced biological functions in vitro.

### Increased recruitment of neutrophils to PSM induced peritonitis in LCAT deficient mice

Previous studies have shown that synthetic PSMs can induce sterile inflammation and recruit neutrophils into a mouse air-pouch model^13^. To pinpoint the role of HDL in protection against PSM-induced inflammation, WT and LCAT^−/−^ mice were subjected to PSM-induced sterile peritonitis. Mice were injected with synthetic PSMα3 intraperitoneally and the peritoneal cells were monitored using an automated hemocytometer.

**Figure 3:**
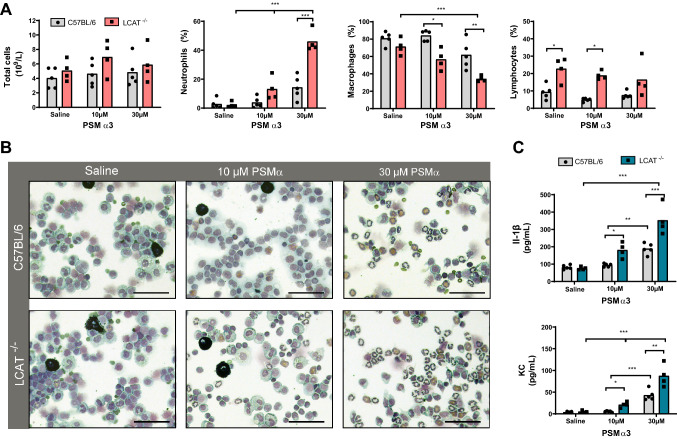
LCAT deficiency enhances neutrophil influx in murine PSM-induced peritonitis model. Peritonitis was induced by injecting 1 mL of saline or 10 µM or 30 µM filter-sterilized PSMα3. Peritoneal leukocytes were harvested and analyzed 6 h after PSM injection, using a hematology Sysmex XT-2000iV analyzer and the total number of cells was quantified (**A**) and the percentage neutrophils, macrophages and lymphocytes was determined. (**B**) Photomicrographs of representative diff-quick stained cytospins of peritoneal cells. Original magnification × 200, bars indicate 50 µm. (**C**) Plasma cytokine levels of IL-1β and KC. Each dot represents an individual mouse, data compiled from 2 individual experiments. * indicates statistical significance of p < 0.05, ** p < 0.01 and *** p < 0.001. One-way ANOVA.

Total numbers of resident peritoneal cells were comparable between WT and LCAT^−/−^ mice following saline control injection and injection of PSMα3 at both 10 μM and 30 μM (Fig. 3A). Interestingly, drastic changes occurred in the composition of peritoneal immune cells of the LCAT^−/−^ mice. PSMα3 treatment resulted in an influx of neutrophils into the peritoneal cavity, which was significantly exacerbated in LCAT^−/−^ mice compared to WT mice. This influx of neutrophils was accompanied by a decrease in peritoneal macrophages in LCAT^−/−^ mice after PSMα3 injection (Fig. 3A). In LCAT^−/−^ mice, there was also an increased proportion of lymphocytes in the peritoneal cavity in both saline and 10 μM PSMα3 treatment (Fig. 1A). Notably, the increase in neutrophil recruitment was less pronounced in the WT control animals and no significant reduction could be observed in peritoneal macrophages, indicating that HDL present in WT mice decreased PSM-induced inflammation.

This change in cellular composition of the peritoneal lavage fluid could be further substantiated with cytospin preparations. Again, large mononuclear cells (macrophages) were the predominant cell type in the lavages of saline-injected control mice. PSM injection in LCAT^−/−^ mice showed a clear decrease in mononuclear cells and a notable increase in ring-shaped nucleated cells (neutrophils) in the peritoneal lavage of PSMα3-treated mice (Fig. 3B). In contrast, in WT animals PSM injection led to a less pronounced change in peritoneal cellular composition. To explain the observed phenotype, the plasma of PSMα3-injected mice was analyzed for the proinflammatory cytokines IL-1β and KC, the mouse equivalent of human IL-8. Levels of plasma IL-1β and KC showed a more pronounced increase in LCAT^−/−^ mice, potentially explaining the enhanced recruitment of neutrophils to the peritoneum (Fig. 3C). Importantly, when washed peritoneal cells (these conditions were devoid of lipoproteins) were stimulated with PSMα3 in vitro*,* no difference was observed in cytokine secretion in the culture medium (Supplementary Fig. 2). Altogether, these results clearly indicate that the biological function of PSMs could be inhibited by HDL in vivo and consequently dampening the inflammatory response.

### MRSA is more virulent during bloodstream infections in mice deficient for HDL

**Figure 4:**
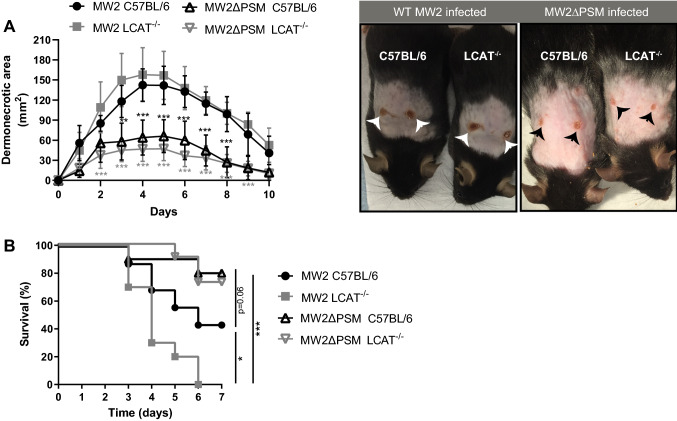
HDL protects against PSM mediated staphylococcal virulence during bloodstream infections, but not during skin infection. (**A**) C57Bl/6 and LCAT^−/−^ mice were infected with *S. aureus* MW2 WT or MW2ΔPSM (MW2Δ*α,β,hld* ) by subcutaneous injection, and developing skin infection was observed. The size of dermonecrotic lesions was measured daily. Mice were infected with 5 × 10^6^ CFU MRSA (n = 10 animals per group). ***, P < 0.001; **, P < 0.01; 2-way ANOVA with Bonferroni post-tests (**B**) Bacteremia model, survival curve. 8 × 10^7^ CFUs of live *S. aureus* MW2 or isogenic MW2ΔPSM were injected into the tail veins of C57Bl/6 and LCAT^−/−^ mice (n = 10–16 animals per group). Survival curves were monitored for statistical significance by the log-rank test*, P < 0.05 ***, P < 0.0001.

Out of all types of staphylococcal disease, skin infections are the most common and in North America most cases are attributed to MRSA strain infections^29^. When a clinically important strain of CA-MRSA was injected subcutaneously into mice, the WT MW2 caused a pronounced skin infection, associated with a gradual development of dermonecrosis (Fig. 4A). This virulent process of MRSA and was not significantly different in LCAT^−/−^ mice, indicating that during skin pathogenesis HDL does not play a significant role in host defense (Fig. 4A). Nevertheless, PSMs were indeed important during skin infections, as shown before^9^, because an isogenic PSM mutant, deficient for all PSM-encoding loci was significantly attenuated in promoting dermonecrosis (Fig. 4A). In PSM mutant infected mice, necrotic lesions developed slower, and never reached the extent observed in the WT MRSA strain (Fig. 4A).

Though there was no protective role observed for HDL during staphylococcal skin infections, the finding that PSMs led to stronger induction of proinflammatory cytokines in HDL-deficient LCAT^−/−^ animals compared to WT mice raised the question if PSMs produced during bloodstream MRSA infections are functionally inhibited by HDL. Intravenous infection of WT and LCAT^−/−^ mice with MRSA led to significant increased mortality in the LCAT^−/−^ mice (Fig. 4B). In LCAT^−/−^ mice all the infected animals succumbed to WT MRSA, whereas 40% of WT mice survived the bloodstream infection (Fig. 4B). In stark contrast, when the experiment was repeated using a PSM mutant, the difference in mortality between WT and LCAT^−/−^ mice was diminished (Fig. 4B). Of note, in accordance with literature^9,12,16,30^, we observed that even in WT animals there was a close to significant (*p* = 0.06) reduction in virulence between the WT MRSA strain and the PSM mutant. Nevertheless, the reduction in virulence of the PSM deficient strain was much more pronounced in LCAT^−/−^ mice (*p* = 0.0001), showing that HDL has a vital role in protection against intravenous infections caused by PSM producing *S. aureus*.

### Plasma derived from patients with impaired ABCA1 function do not neutralize PSMs

**Figure 5:**
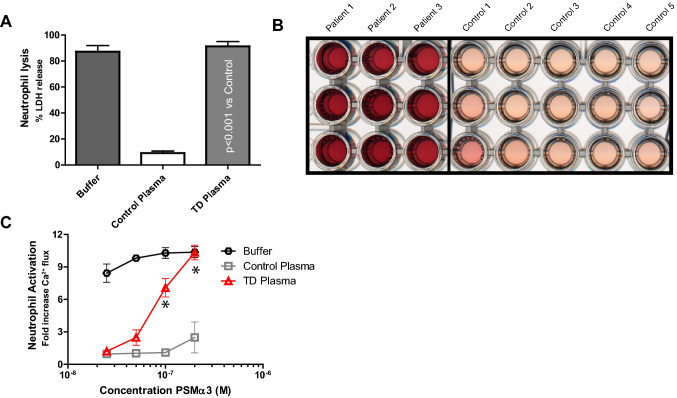
Reduced protection against PSM-induced neutrophil activation and lysis by plasma from Tangier patients. (**A**) PSMα3 (10 µM) induced human neutrophil lysis after preincubation with 0.5% plasma from three different Tangier disease (TD) patients and five healthy control donors. Neutrophil lysis was measured through LDH release. (**B**) Representative photograph of 1 out of 3 neutrophil lysis experiments, as explained under A. (**C**) Calcium mobilization of HL-60/FPR2 stimulated with (25 – 200 nm PSMα3) with and without pretreatment with 0.01% Tangier disease (TD) plasma or control plasma. Calcium mobilization was measured by flow cytometry. * Indicate statistical significance of p < 0.01 One-way ANOVA. Data represent means ± SEM of 3 independent experiments.

Homozygous carriers of ABCA1 variants are characterised by HDL deficiency. This very rare form of hypobetalipoproteinemia is named Tangier disease (TD) and TD patients present with enlarged lipid-laden tonsils^31^. Therefore, we investigated whether plasma of these patients could similarly decrease the potential to protect against staphylococcal PSMs. Indeed, plasma from 3 TD patients did not protect against PSMα3-induced neutrophil lysis in vitro, in contrast to control plasma samples (Fig. 5A,B). In addition, plasma of TD patients was significantly less effective in neutralization of PSMα3-mediated calcium fluxes in FPR2-HL60 cells (Fig. 5C). Thus, HDL-deficient plasma of TD patients fails to protect against PSM-mediated neutrophil activation and lysis. Given the very low incidence of Tangier disease, it is impossible to asses whether TD patients are at increased risk factor for staphylococcal disease compared to non ABCA1 variant carriers/controls.

## Discussion

The number of infections by *S. aureus* remains high, especially infections caused by methicillin-resistant *S. aureus* (MRSA) strains. Nevertheless, infections occur only in a small percentage of individuals who are colonized by *S. aureus*, which means that the innate immune system efficiently controls staphylococcal infections. In recent years it has become clear that besides the well-known players, such as immune cells, complement, antimicrobial peptides, and chelating proteins, lipids and lipoproteins participate in innate immunity against invading microorganisms as well. Multiple studies have shown that lipoproteins serve as scavengers by binding microbes or cell wall components derived from microorganisms^32^. For instance, lipoteichoic acid (LTA) present in the cell wall of staphylococci, or lipopolysaccharide (LPS), the major outer membrane component of Gram-negative bacteria, bind mainly to HDL within human blood^33,34^. This binding to HDL neutralizes the pro-inflammatory effects of LPS or LTA, inhibiting their interaction with Toll-like receptors and activation of immune cells^35^. Furthermore, apolipoprotein B (apoB) containing lipoproteins can interfere in the communication between staphylococcal cells^36^. Accumulation of secreted autoinducing peptide (AIP) is normally sensed by the accessory gene regulator (*agr*) system and triggers the secretion of toxins and extracellular enzymes. However, apoB in VLDL and LDL present in the blood can sequester AIP and thereby disable staphylococcal quorum sensing^36^. Moreover, mice lacking plasma apoB are more susceptible to invasive staphylococcal infections, highlighting that apoB is an important innate defense effector against *S. aureus*^36,37^. We previously reported that HDL and LDL present in the blood specifically inhibit staphylococcal PSMs. When human serum was spiked with PSMs and different lipoprotein subsets were analyzed for the presence of PSMs, approximately 80% of the spiked PSMs were found in the HDL-containing fractions, whereas approximately 15% and 5% of PSM was recovered in LDL- and VLDL-containing fractions, respectively. Thus, within human blood, HDL is the most potent inhibitor of PSMs^17^.

In the current study, we extended on previous work and showed that in vitro and in vivo that murine HDL is an innate barrier for staphylococcal virulence by neutralizing the important PSM toxins. HDL deficiency in LCAT^−/−^ and ABCA1^−/−^ mice resulted in a dramatic reduction in the protection against PSM-induced neutrophil lysis, again pinpointing HDL as a major lipoprotein responsible for PSM neutralization. In addition, we showed using PSM-induced sterile peritonitis in HDL deficient LCAT^−/−^ mice that PSMs displayed increased lysis of peritoneal macrophages, resulted in enhanced neutrophil influx and more pro-inflammatory cytokine production, clearly showing that in vivo neutralization of PSMs occurs by HDL particles. This implicates that tissue lipoprotein levels are sufficient for uptake of PSMs and thereby dampening of the host immune response. In line with this, Sigel et al*.* showed an enhanced pro-inflammatory cytokine response after *S. aureus* challenge in mice where lipoprotein secretion was pharmacologically inhibited^38^. It is tempting to speculate that this enhanced pro-inflammatory cytokine response is caused by the lack of PSM neutralization at these low lipoprotein levels.

Dampening this pro-inflammatory response by HDL could have multiple underlying molecular mechanisms. First, in our work we show that direct PSM-induced activation of neutrophils is inhibited by HDL, but this is likely similar for all FPR2 expressing immune cells. Second, work from Peschel et al*.* showed that PSMs induced the release of lipoproteins from the bacterial surface resulting in an enhanced pro-inflammatory response through activation of TLR2 on immune cells^16^. HDL could potentially interfere with this process and reduce the number of lipoproteins released from the staphylococci thereby dampening the proinflammatory response. Third, lysis of host immune cells results in a pronounced pro-inflammatory response resulting in ample neutrophil recruitment^39^. Our sterile peritonitis model shows HDL-mediated protection of peritoneal macrophages from PSM-induced lysis, which could also account for the reduction of neutrophil recruitment and pro-inflammatory cytokine release observed. Most likely, HDL could influence the proinflammatory processes through each of those above-mentioned mechanisms and would be very interesting to elucidate the contribution of each of those mechanisms in future studies. Nevertheless, here we show a profound role of HDL-dependent PSM neutralization in sterile inflammation.

Multiple studies, including this one, have found a profound role for PSMs in promoting staphylococcal pathogenesis in experimental infection models^9,12,16,30^. Although, *S. aureus* can secrete PSMs in large quantities^8,9^, during bloodstream infection it is likely that high levels of HDL will rapidly soak up any extracellular PSMs and neutralize this staphylococcal virulence factor^17^. Indeed, our intravenous infection study showed significantly enhanced PSM-mediated mortality in LCAT^−/−^ mice compared to WT mice, clearly showing protection by HDL against staphylococcal PSMs in this model. Interestingly, this difference was not observed during skin infection, suggesting that the HDL concentration in skin may not be high enough to neutralize PSMs. Furthermore, when the staphylococcal abscess develops the bacterial community is shielded by fibrin deposits and form a pseudo capsule which might be impermeable for lipoproteins^40^. Alternatively, it could be that HDL indeed neutralizes secreted PSMs during skin lesions, but that the primary function of PSMs is not to activate and lyse immune cells in the extracellular tissue environment as previously suggested^9^.

There is an increasing body of evidence that intracellular production of PSMs after staphylococcal ingestion into immune cells plays an important role during pathogenesis^41^. Importantly, within this intracellular niche, PSM production cannot be reached for neutralization by lipoproteins. This could also explain the residual PSM-mediated reduction (p = 0.06) of virulence in WT mice showing that when high levels of HDL are present PSMs could still mediate virulence. This is in accordance with multiple other studies^9,30^.

Using intravital microscopy in live mice it was shown that during bloodstream infections *S. aureus* is captured by Kupffer cells in the liver and these cells form an important bottleneck for further dissemination^42–44^. However, a minority of the staphylococci overcome the Kupffer cells antimicrobial defenses. Over time, the Kupffer cells lyse, releasing bacteria into the circulation and/or peritoneal cavity, enabling dissemination to other organs such as the kidneys^45^. During these stages, staphylococci infect multiple phagocytic cell types and could even use host cells as transport to disseminate from the site of infection^46–48^. Therefore, we think that PSM function might be an important toxin in mediating the escape from the phagosome and lysis of the host cell^41^. While in the phagolysosome of immune cells, *S. aureus* needs to adopt a more virulent lifestyle, most likely dependent on the intracellular activation of two-component systems such as the Agr system^49^. The concentration of the quorum sensing pheromone AIP can reach the critical concentration necessary for Agr activation inside host cells^50,51^. It was also demonstrated that the stringent response, characterized by the rapid synthesis of (p)ppGpp as messenger of environmental stress conditions, seems to precede the quorum sensing mechanism^52^. Both systems appear to be crucial for up-regulating PSMs in the phagosome and thereby contribute to the lysis of phagocytes after phagocytosis, enabling the subsequent escape of the staphylococci. However, among different *S. aureus* strains several mechanisms, instead of a single virulence factor, play a role in staphylococcal escape after phagocytosis; and different toxins may cause escape in different cell types.

Overall, the current study shows that HDL, an important plasma component for lipid metabolism, can functionally neutralize staphylococcal PSMs during infection. These findings emphasize another important role of HDL in host defense by functioning as a sink for the important staphylococcal virulence factor PSMs.

## Material and methods

### Ethics statement

Informed written consent was obtained from all consent was obtained from all participants in accordance with the Declaration of Helsinki Approval was obtained from the medical ethics committees of the University Medical Center Utrecht (Utrecht, The Netherlands), the Academic Medical Center (Amsterdam, The Netherlands) and the University of Calgary (Calgary, Alberta, Canada).

### Reagents

PSM peptides were synthesized with the published sequences^9^ by Genscript at 95% purity. PSMα1 (MGIIAGIIKVIKSLIEQFTGK), PSMα2 (MGIIAGIIKFIKGLIEKFTGK), PSMα3 (MEFVAKLFKFFKDLLGKFLGNN), PSMα4 (MAIVGTIIKIIKAIIDIFAK), and δ-toxin (MAQDIISTISDLVKWIIDTVNKFTKK) were all synthesized with an N-terminal formyl methionine residue. Peptide stocks were prepared at 2 mM and dissolved in H_2_O except PSMα4, which was dissolved in 50% (v/v) MeOH/H_2_0.

### Mice

LCAT^−/−^ mice were a kind gift from Dr. Cheryl L. Wellington^49^, and ABCA1^−/−^mice were kindly provided by Dr. G. Chimini^25^. C57Bl/6 mice were obtained from the Jackson Laboratory as mating pairs, and bred in Gorlaeus Laboratories (Leiden, The Netherlands) and at the University of Calgary. Mice were maintained on a sterilized regular chow diet, containing 4.3% (w/w) fat and no cholesterol (RM3, Special Diet Services, Witham, UK). Animal experiments were carried out with male adult 8–12-wk-old mice and all experimental animal protocols were approved by the University of Calgary Animal Care Committee and were in compliance with the Canadian Council for Animal Care Guidelines (protocol nr. AC16-0148) Animal experiments that were performed at the Gorlaeus Laboratories of the Leiden/Academic Center for Drug Research in accordance with the National Laws (ID 04081.1) and performed in accordance with the ARRIVE guidelines and the Directive 2010/63/EU of the European Parliament. For the peritonitis model LCAT^−/−^ and age matched C57BL/6 received an intraperitoneal injection with 1 mL of 10 µM or 30 µM PSMα3 or saline only. Six hours after PSMα3 exposure mice were euthanized and blood was drawn into EDTA through orbital extraction. The peritoneal cavity of the mice was lavaged with 10 mL cold PBS to collect peritoneal leukocytes. Leukocyte quantification of peritoneal lavage samples and blood was performed using an automated Sysmex XT-2000iV Veterinary Heamatology analyzer (Sysmex Corporation). Corresponding samples were cytospun for manual confirmation and cells were stained with Diff-Quick (Baxter) to visualize leukocyte accumulation in the peritoneum. Plasma was isolated by centrifugation of whole blood at 4000 rpm for 10 min at 4 °C. KC and IL-1B cytokine levels were measured by ELISAS (Invitrogen) according to the manufacture’s protocols.

### Infection studies

*Staphylococcus aureus* strains MW2 and its isogenic Δαβ*hld* PSM mutant (All psm genes are deleted. Translation of hld is abolished my mutation of the start codon)^30^ was used for all the experiments. Bacteria were grown in Brain Heart Infusion (BHI) media at 37C while shaking. For lethal infection experiments, *S. aureus* strains/mutants were sub-cultured until exponential phase (OD660nm 1.0) washed with saline once, resuspended in saline and injected intravenously in the tail vein of C57BL/6 or LCAT^−/−^ mice at 8 × 10^7^ CFU in 200 mL. A murine sepsis score system was introduced to monitor the mice based on their appearance, level of consciousness, activity, response to stimuli, eyes, respiration rate and quality (from 0 to 4 points for each criteria). An overall score of 18 or a score of 4 in any of the following criteria was considered as a humane endpoint: level of consciousness, activity, response to stimulus, respiration rate and respiration quality^53^. Alternatively, a murine skin infection model was used. Dorsums of of C57BL/6 or LCAT^−/−^ mice were shaved, and wiped with alcohol pads, 30ul containing 10^7^ CFU bacteria, was injected subcutaneously through an insulin syringe. Developing lesions were photographed daily, and the area was measured with FIJI software.

### Cells

HL-60 cells stably transfected with the FPR2 (HL-60/FPR2), were kindly provided by F. Boulay (Laboratoire Biochimie et Biophysique des Systemes Integres, Grenoble, France). Cells were cultured in RPMI-1640 supplemented with 10% fetal bovine serum, 2 µM L-glutamine, 100 units/ml penicillin, 100 μg/ml streptomycin, and 600 μg/ml G418. Human neutrophils were isolated from venous heparinized blood by means of the Ficoll-Histopaque gradient method as described^54^. Mouse neutrophils were isolated as described^39^.

### Plasma preparations

Freshly drawn venous blood from healthy controls as well as TD patients was collected after overnight fasting into 0.1 volume 130 mmol/L trisodium citrate. Plasma was obtained by centrifugation and stored until use at − 70 °C. Plasma was heat-inactivated by heating at 56 °C for 20 min.

### Calcium mobilization in human neutrophils and HL-60 cells

Calcium mobilization with isolated human neutrophils and HL-60/FPR2 cells was performed as previously described^17^. For this purpose, cells (5 × 10^6^ cells/ml) were labeled with 2 μM Fluo-3-AM, protected from light, with gentle agitation, for 20 min at room temperature. The cells were washed and resuspended in RPMI-HSA (without FCS) to 5 × 10^6^ cells/ml. Stimuli were prepared by incubating 25 µl of 10 times concentrated agonist with 25 µl 10 times concentrated heat inactivated human or mice plasma or buffer for 30 min at room temperature. Before stimulation, cells were diluted to 1 × 10^6^ cells/ml in a volume of 200 μl. The background fluorescence level for Fluo-3 was monitored for 8 s after which 50 μl of pre-incubated stimulus was added. The sample tube was rapidly placed back to the sample holder and the fluorescence measurement continued up to 52 s. Cells were gated based on scatter parameters to exclude cell debris and the mean fluorescence value at basal level was subtracted from the value at peak level (at 30 s). The different fluorescent values were expressed as percentage of the maximal response for each individual stimulus. Alternatively, various concentrations of culture supernatants or synthetic PSM, pre-incubated with various concentrations human or mice plasma for 15 min, were added to Fluo-3-labeled HL-60/FPR2 cells followed by flow cytometry.

### Neutrophil lysis assay

Lysis of human neutrophils by synthetic PSMs was measured as previously described^17^. PSMs were pre-incubated with different concentrations of human or mice plasma for 10 min at room temperature. Pre-treated supernatants were transferred to a 96-wells microtiter plate (Nunc) containing 3 × 10^6^ neutrophils in a total volume of 100 µl RPMI-HSA and incubated for 15 min at 37 °C. Neutrophil lysis was determined by release of lactate dehydrogenase (LDH) using the CytoTox 96 Non-Radioactive Cytotoxicity kit (Promega) according to the manufacture’s protocols.

### Statistics

Data were analyzed by Graph Pad Prism 5 software using two-tailed Student's t-test or a one-way ANOVA using an appropriate posttest.

## Supplementary Information

Supplementary Information.
