# Supplementary material for: High density lipoproteins mediate in vivo protection against staphylococcal phenol-soluble modulins
Source: Sci Rep. 2021 Jul 28;11:15357. doi: 10.1038/s41598-021-94651-1 (PMC8319287; doi:10.1038/s41598-021-94651-1)
Supplement: Supplementary file 1 — Supplementary Information. [file 41598_2021_94651_MOESM1_ESM.pdf]

Supplementary information

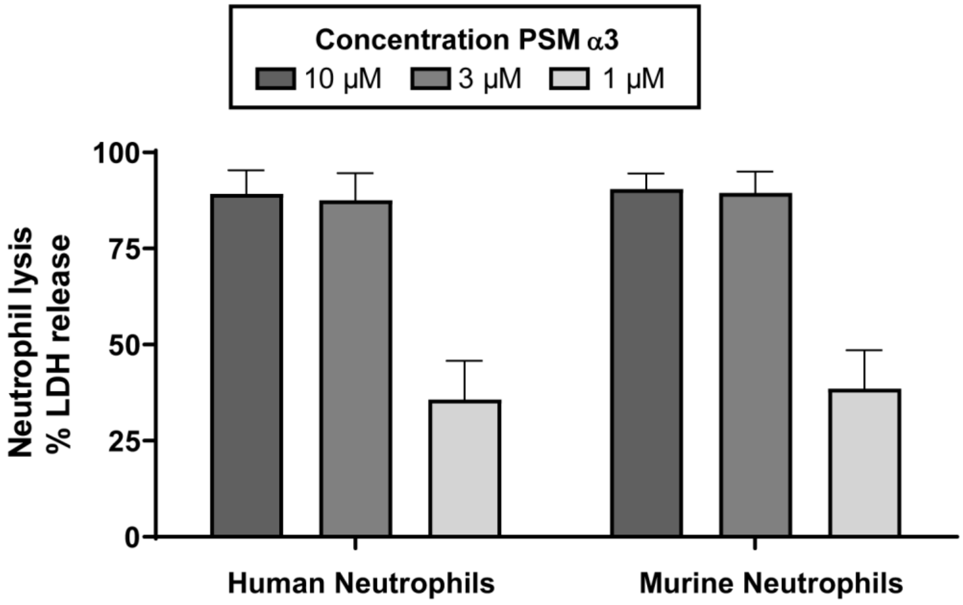

S Figure 1: No difference in PSM induced lyses between human and murine neutrophils. Dose-dependent neutrophil lysis induced by synthetic PSMα3. Neutrophils were isolated form healthy human donors or murine bone marrow. Neutrophil lysis was measured via LDH release. Data represent means ± SEM of three independent experiments.

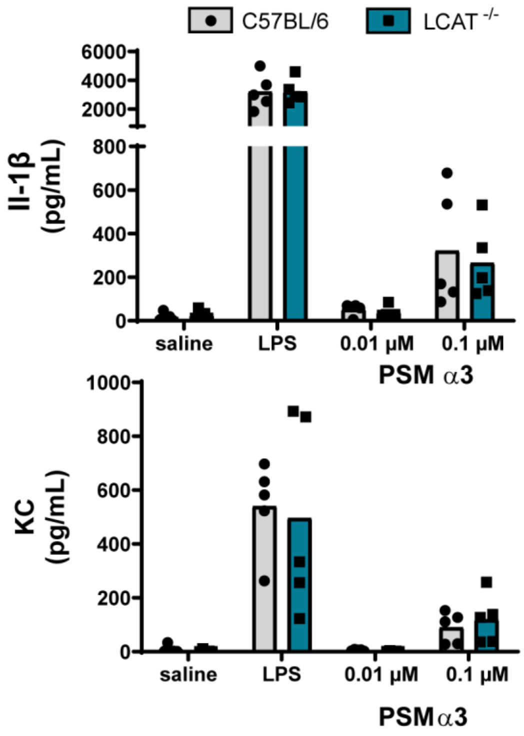

S Figure 2: Peritoneal cells isolated from C57BL/6 or LCAT-/- mice produce equal amount of pro-inflammatory cytokines in response to stimulation with PSM. *In vitro* stimulation of peritoneal lavage cells isolated from C57BL/6 or LCAT$^{-/-}$ mice with LPS (50ng/ml) or synthetic PSMα3. levels of IL-1β and KC were measured by ELISA. Each dot represents an individual mouse, data compiled from 2 individual experiments.
